# FlyRNAi.org 2025 update—expanded resources for new technologies and species

**DOI:** 10.1093/nar/gkae917

**Published:** 2024-10-22

**Authors:** Yanhui Hu, Aram Comjean, Jonathan Rodiger, Weihang Chen, Ah-Ram Kim, Mujeeb Qadiri, Chenxi Gao, Jonathan Zirin, Stephanie E Mohr, Norbert Perrimon

**Affiliations:** D epartment of Genetics, Blavatnik Institute, Harvard Medical School, 77 Avenue Louis Pasteur, Boston, MA 02115, USA; Drosophila RNAi Screening Center, Harvard Medical School, 77 Avenue Louis Pasteur, Boston, MA 02115, USA; D epartment of Genetics, Blavatnik Institute, Harvard Medical School, 77 Avenue Louis Pasteur, Boston, MA 02115, USA; Drosophila RNAi Screening Center, Harvard Medical School, 77 Avenue Louis Pasteur, Boston, MA 02115, USA; D epartment of Genetics, Blavatnik Institute, Harvard Medical School, 77 Avenue Louis Pasteur, Boston, MA 02115, USA; Drosophila RNAi Screening Center, Harvard Medical School, 77 Avenue Louis Pasteur, Boston, MA 02115, USA; LifeMine Therapeutics, 30 Acorn Park Dr, Cambridge, MA 02140, USA; D epartment of Genetics, Blavatnik Institute, Harvard Medical School, 77 Avenue Louis Pasteur, Boston, MA 02115, USA; Drosophila RNAi Screening Center, Harvard Medical School, 77 Avenue Louis Pasteur, Boston, MA 02115, USA; D epartment of Genetics, Blavatnik Institute, Harvard Medical School, 77 Avenue Louis Pasteur, Boston, MA 02115, USA; Drosophila RNAi Screening Center, Harvard Medical School, 77 Avenue Louis Pasteur, Boston, MA 02115, USA; D epartment of Genetics, Blavatnik Institute, Harvard Medical School, 77 Avenue Louis Pasteur, Boston, MA 02115, USA; Drosophila RNAi Screening Center, Harvard Medical School, 77 Avenue Louis Pasteur, Boston, MA 02115, USA; D epartment of Genetics, Blavatnik Institute, Harvard Medical School, 77 Avenue Louis Pasteur, Boston, MA 02115, USA; Drosophila RNAi Screening Center, Harvard Medical School, 77 Avenue Louis Pasteur, Boston, MA 02115, USA; D epartment of Genetics, Blavatnik Institute, Harvard Medical School, 77 Avenue Louis Pasteur, Boston, MA 02115, USA; Drosophila RNAi Screening Center, Harvard Medical School, 77 Avenue Louis Pasteur, Boston, MA 02115, USA; D epartment of Genetics, Blavatnik Institute, Harvard Medical School, 77 Avenue Louis Pasteur, Boston, MA 02115, USA; Drosophila RNAi Screening Center, Harvard Medical School, 77 Avenue Louis Pasteur, Boston, MA 02115, USA; D epartment of Genetics, Blavatnik Institute, Harvard Medical School, 77 Avenue Louis Pasteur, Boston, MA 02115, USA; Drosophila RNAi Screening Center, Harvard Medical School, 77 Avenue Louis Pasteur, Boston, MA 02115, USA; Howard Hughes Medical Institute, 77 Avenue Louis Pasteur, Boston, MA 02115, USA

## Abstract

The design, analysis and mining of large-scale ‘omics studies with the goal of advancing biological and biomedical understanding require use of a range of bioinformatics tools, including approaches tailored to needs specific to a given species and/or technology. The FlyRNAi database at the Drosophila RNAi Screening Center and Transgenic RNAi Project (DRSC/TRiP) Functional Genomics Resources (https://fgr.hms.harvard.edu/tools) supports an increasingly broad group of technologies and species. Recently, for example, we expanded the database to include additional new data-centric resources that facilitate mining and analysis of single-cell transcriptomics. In addition, we have applied our approaches to CRISPR reagent and gene-centric bioinformatics approaches in *Drosophila* to arthropod vectors of infectious diseases. Building on our previous comprehensive reports on the FlyRNAi database, here we focus on new and updated resources with a primary focus on data-centric tools. Altogether, our suite of online resources supports various stages of functional genomics studies for *Drosophila* and other arthropods, and facilitate a wide range of reagent design, analysis, data mining and analysis approaches by biologists and biomedical experts studying *Drosophila*, other common genetic model species, arthropod vectors and/or human biology.

## Graphical Abstract

**Graphical Abstract:**
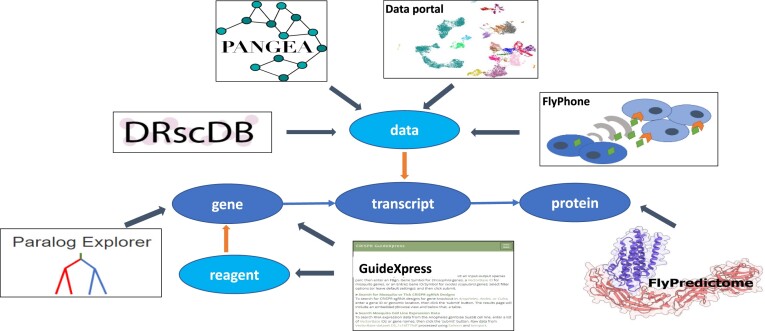

## Introduction

Our group, known for years as the Drosophila RNAi Screening Center (DRSC) and also known as the DRSC/TRiP Functional Genomics Resources and Drosophila Research and Screening Center-Biomedical Technology Research Resource (DRSC-BTRR), founded the FlyRNAi database in 2004. At that time, the focus of the resource was on RNAi-based cell screening technology in *Drosophila* (1–3). Since then, however, our facility and the corresponding FlyRNAi resource have broadened and evolved, in response for example to (i) the development of new technologies such as CRISPR-based gene perturbation (4), (ii) the need for integration of information from multiple model species and humans (5–7) and (iii) the need for resources supporting work in non-model arthropods (8). We report here on our most recent efforts, which are relevant to new technologies including single-cell RNAseq and AI-driven protein structure predictions, and help meet the needs of researchers studying arthropods of increasing public health concern, namely, mosquito and tick vectors of human diseases.

The design, analysis and mining of large-scale ‘omics studies require the application of a range of specialized bioinformatics tools, including approaches focused on reagent design, data analysis and visualization, and data integration and mining. In many cases, these approaches must be tailored to needs dictated by the species under study, the experimental technology being used and the goals of the research study. Our group built the FlyRNAi database and a corresponding suite of online resources (https://fgr.hms.harvard.edu/tools) with the initial goal of helping researchers and others meet these specialized needs for *Drosophila* research and other applications. Broadly speaking, these resources make it possible for researchers to design reagents and analyze, integrate, and mine data, as introduced and updated in previous reports (2–4) and summarized in Table 1. Overall, we strive to generate resources that (i) complement what is already available from model organism databases such as FlyBase (9) or the Alliance for Genome Resources (10), (ii) are guided by the needs of researchers engaged in ‘omics studies such as high-throughput cell screens in *Drosophila* cells or *in vivo* (11–14) or in mosquito cells (8) and (iii) help biological and biomedical researchers make use of the wealth of information available from *Drosophila* and other species to design and interpret the outcomes of ‘omics and other high- or low-throughput studies.

**Table 1.** Major DRSC bioinformatics tools and resources built on the FlyRNAi database

Focus	Resource	Number of species supported	Resource function and URL
**Genes and Data**	**Paralog Explorer** (20)	**5**	**Search paralogs and related data https://www.flyrnai.org/tools/paralogs/web/**
Genes	BioLitMine (44)	10	Mine the published literature https://www.flyrnai.org/tools/biolitmine/web/
Genes	Gene2Function (5)	9	Compare genes across multiple species https://www.gene2function.org/search/web/
Genes	GLAD (45)	1 (*Drosophila*)	Retrieve gene group annotations https://www.flyrnai.org/tools/glad/web/
Genes	DIOPT (6)	12*	Search orthologs and paralogs https://www.flyrnai.org/DIOPT
Genes	DIOPT-DIST (6)	12	Search disease-related orthologs https://www.flyrnai.org/diopt-dist
**Genes and Reagents**	**GuideXpress**(8)	**8 (mosquitos, tick, *Drosophila*, others)**	**Search orthologs and sgRNA designs https://www.flyrnai.org/tools/fly2mosquito/web/**
Reagents	Find CRISPR Tool (46)	1 (*Drosophila*)	Search sgRNA designs (for reference wild-type alleles) https://www.flyrnai.org/crispr3/web/
Reagents	SNP CRISPR (47)	9*	Search sgRNA designs (for mutant alleles) https://www.flyrnai.org/tools/snp_crispr/web/
Reagents	UP-TORR (48)	4	Search *Drosophila* RNAi reagents (cell-based or *in vivo*) https://fgr.hms.harvard.edu/up-torr
Reagents	FlyPrimerBank (49)	1 (*Drosophila*)	Search qPCR primer designs https://www.flyrnai.org/flyprimerbank
Reagents	SnapDragon (1)	1 (*Drosophila*)	Design long double-stranded RNAs (dsRNAs) https://www.flyrnai.org/snapdragon
Reagents and Data	RSVP (50)	1 (*Drosophila*)	Search *in vivo* RNAi and CRISPR sgRNA stocks and data https://www.flyrnai.org/cgi-bin/RSVP_search.pl
**Data**	**FlyPredictome** (18)	**1 (*Drosophila*)**	**Mine predicted protein-protein interactions https://www.flyrnai.org/tools/fly_predictome**
**Data**	**PANGEA** (17)	**6**	**Perform gene set enrichment analysis (GSEA) https://www.flyrnai.org/tools/pangea**
**Data**	**FlyPhoneDB** (16)	1 (*Drosophila*) *	**Analyze scRNA-seq to predict cell-cell communication https://www.flyrnai.org/tools/fly_phone**
**Data**	**DRscDB** (15)	**5**	**Mine and compare scRNA-seq data https://www.flyrnai.org/tools/single_cell**
Data	Single Cell Portal (22–26)	1 (*Drosophila*)	Mine in-house scRNA-seq datasets https://www.flyrnai.org/scRNA/
Data	PathOn (51)	1 (*Drosophila*)	Analyze RNA-seq data for signaling pathways https://www.flyrnai.org/tools/pathon/web/
Data	iProteinDB (41)	7	Mine PTM data https://www.flyrnai.org/tools/iproteindb/web/
Data	MIST (27)	10	Mine interaction data https://fgrtools.hms.harvard.edu/MIST/
Data	DGET (52)	1 (*Drosophila*)	Mine bulk-RNA-seq data https://www.flyrnai.org/tools/dget/web/
Data	COMPLEAT (53)	3	Protein complexes enrichment tool https://www.flyrnai.org/compleat/

Newly added tools are in bold text.

*Indicates availability of stand-alone code or a pipeline that can be run for additional species.

Abbreviations: PTM, post-translational modifications; RNAi, RNA interference; sgRNA, single guide RNA.

For resources newly added or updated since our last update, our main focus was in the area of data-centric tools. For example, new features to current resources make it possible to compare data from multiple experimental single cell RNA-Sequencing (scRNA-Seq) datasets, allowing researchers to mine datasets across genotypes or conditions. In addition, we implemented new data-centric analysis tools. These include DRscDB (15), a comprehensive repository for published scRNA-seq datasets for *Drosophila* and relevant datasets from human and other model organisms, and FlyPhoneDB (16), which supports the generation of new hypotheses regarding cell-cell communication based on scRNA-seq data. Furthermore, we developed PANGEA (17) to support gene-set enrichment analysis (GSEA) that is specifically tailored for analysis of gene lists from common genetic model organisms. FlyPredictome (18) is the most recent addition to FlyRNAi. With this resource, users can view predicted protein-protein interactions based on AlphaFold Multimer (AFM) (19) analysis, primarily for *Drosophila* but also for some human proteins.

Although data-centric resources were our main focus, they have not been our only focus. For example, we have also actively improved and expanded our gene-centric tools. Paralog Explorer (20) applies our integrative DIOPT approach (6) to identification of putative paralogs and integrates relevant public datasets, such as RNA expression and interaction datasets. In addition, we implemented a local pipeline for our DIOPT ortholog resource to help predict orthologs for under-studied species. Last but not least, we launched GuideExpress, building on our experience and infrastructure in functional genomics and other topics to provide gene-, data-, and reagent-centric support for the study of mosquito vectors of infectious diseases (8) and have since added support for *Ixodes scapularis*, a tick vector of Lyme and other diseases.

## Overview and use case for data-centric resources

### Data-centric resources for high-resolution transcriptomics data

**Figure 1.**
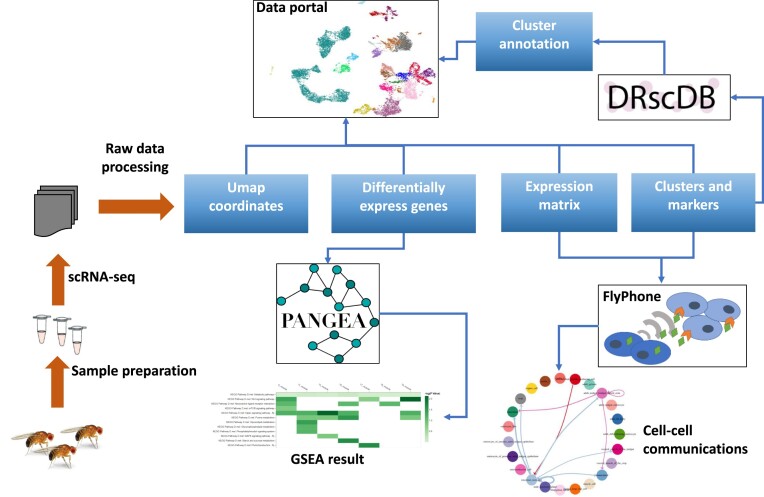
Relationships among DRSC resources relevant to single cell or single nucleus RNA sequencing (scRNA-Seq). Following experimental steps and raw data processing, processed data from scRNA-seq studies can be further analyzed. For example, marker genes from each cluster can be compared with marker genes from other studies for cell type assignment using DRscDB. FlyPhone can be used to predict information flow based on expression of ligands, receptors, and effectors genes in various cell types. In addition, differentially expressed genes can be analyzed by PANGEA to understand the underlining biology of the perturbation while full data can be easily mined at DRSC scRNA-seq data portal. A detailed example of how multiple tools can be used to analyze a dataset is provided in Supplementary Figure S1.

Single-cell and single-nucleus RNA sequencing (scRNA-Seq) technology allows researchers to build atlases of cell types and states, including in wild-type or perturbed conditions. Mining of scRNA-seq datasets can lead to new insights and precision for topics such as cell type diversity and complexity, and changes in cell types and gene expression in response to treatments. The volume and complexity of scRNA-seq datasets available for common model systems such as *Drosophila* have increased rapidly in recent years, creating a growing need for informatics tools to support analysis, visualization, comparison, and integration of scRNA-seq data. To help meet this need, we developed DRscDB (15) and FlyPhoneDB (16) to support mining, annotation, comparison and analysis of scRNA-seq datasets from *Drosophila* and other species, and expanded the capabilities of our scRNA-seq data portal (Figure 1).

***DRscDB*:** The DRSC single cell RNA-seq database (DRscDB; URL for this and subsequent resources shown in Table 1) centralizes major scRNA-seq datasets generated from various organs of the adult fly and some relevant datasets from human, mouse, and zebrafish studies, making it possible for users to query expression patterns across studies and across species for a given input gene. Different from source databases in which the original data is stored, such as the SCope site of Fly Cell Atlas (21), DRscDB summarizes cluster-level statistics and presents visualizations of cluster-level data and marker calling results using various plots. In addition, DRscDB also facilitates enrichment analysis of cell type-specific marker genes. This helps users identify cell type(s) closely associated with a gene list of interest, making it easier to annotate cell types when new datasets are generated. Thus, DRscDB aids users by supporting both mining of existing scRNA-seq data and annotation of new scRNA-seq datasets.

***FlyPhoneDB*:**We further recognized that information embedded in scRNA-seq datasets can be combined with existing knowledge to build hypotheses regarding signaling from one cell type to another. To support this, we first built the FlyPhone knowledgebase of manually curated ligand-receptor pairs for major signaling pathways in *Drosophila*. We then implemented the FlyPhone Database (FlyPhoneDB) (16) to make it possible for users to evaluate ligand and receptor expression patterns in scRNA-seq datasets and identify potential cell-cell communication events between cell types. This is the first tool analyzing cell-cell communication in *Drosophila* scRNA-seq data while all the other tools developed for this purpose focused on mammalian data with the exception of DanioTalk developed for analyzing zebrafish data.

**Figure 2.**
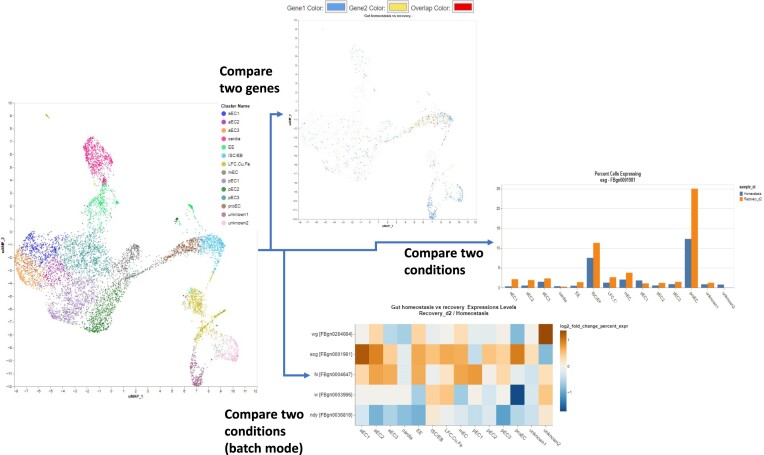
New features in the DRSC scRNA-seq data portal. In its original form, our portal supported visualizations based on a single gene or condition. Our updates added the ability to simultaneously visualize expression of two genes on the UMAP (center top panel). In addition, we added the ability to compare results obtained for scRNA-seq of two samples (e.g. untreated vs treated or wildtype vs mutant). Comparison can be done for a single gene (graphical output comparing the percentage or average expression of input gene in each cell type) or in batch mode (heatmap output).

***scRNA-seq data portal:*** Our scRNA-seq data portal (Single Cell Portal) allows researchers to mine datasets generated by the Perrimon lab. In recent years, we have been adding more datasets (22–26) and features that make data mining more efficient (Figure 2). For example, the new features allow users to compare expression patterns between two input genes within a sample, as well as compare expression patterns between two samples (e.g. different conditions or genotypes) for any input gene. The latter feature supports both searches with a single gene (bar graph output) and ‘batch-mode’ searches with lists of multiple genes (heatmap output).

### Additional new data-centric resources

While DRscDB, FlyPhoneDB, and the scRNA-seq data portal specifically help begin to reveal mechanisms underlying cell states, such as cell-cell communication, we wanted to go further in our support for scRNA-seq and other ‘omic’ data outputs. Thus, with analysis of scRNA-seq and other data types in mind, we developed the PAthway, Network and Gene-set Enrichment Analysis (PANGEA) resource (17), and to build on our past developments in the area of protein-protein interactions (PPIs), such as development of the Molecular Interaction Search Tool (MIST) (27), we added FlyPredictome.

***PANGEA:*** PANGEA is a gene set enrichment analysis (GSEA) tool that allows users to query a knowledgebase we assembled from gene or protein annotations assembled from gene set annotations available from a variety of sources, some of which are unique to *Drosophila*. Our annotations include gene ontology (GO) (28,29), KEGG (30) and Reactome (31) annotations, as well as high-quality curated sets of genes, including ‘gene groups’ (groups of genes related based on protein domains, enzymatic functions, and/or other features), pathways, and phenotypes, that are available from FlyBase (9,32,33) and the Alliance of Genome Resources (10). Notably, as GSEA plays an important role in analysis of other large-scale datasets, the utility of PANGEA extends to other types of ‘omics datasets as well, including proteomics and functional genomics datasets. PANGEA also makes it possible to generate visualizations of enrichment results, including a network view of genes and associated gene sets, making it easy to view what genes drive the enrichment results and what enriched genes are present in multiple gene sets. In addition, PANGEA allows parallel analysis of multiple gene lists and makes it possible for users to compare results by quickly generating heatmap and/or dot plot views. Features of PANGEA such as inclusion of curated gene lists specific to *Drosophila* and generation of various types of visualizations within the platform distinguish PANGEA from other commonly used GSEA resources such as DAVID and WebGestalt (34,35).

***FlyPredictome*:**The application of artificial intelligence (AI) to the challenge of predicting protein structure, e.g. using AlphaFold (36), has radically changed the pace and precision with which protein structures can be predicted. Further, the application of AlphaFold has been extended to prediction of protein complexes using AlphaFold-Multimer (AFM) (19). To add additional support for protein-focused data, we developed a pipeline for evaluating protein-protein interactions (PPIs) using AFM and built FlyPredictome to house the outcomes and associated information (18). In particular, we used AFM to evaluate PPIs in *Drosophila* and human PPI datasets by focusing on Predicted Aligned Error, an indicator of likely interactions, and to predict new interactions to expand PPI networks. At FlyPredictome, users can mine the AFM-based predictions and evaluations, as well as view predicted structures and other information, for more than 100 000 *Drosophila* protein pairs. Databases of AFM outcomes are currently limited. To our knowledge, the only database that is comparable in scale (∼50 000 interactions) is the HumanPredictome database, which is focused on human proteins relevant to genome maintenance and histones (37).

### A use case for data-centric tools

As a use case to demonstrate the utility of our data-centric tools, we present here an analysis of a published dataset using multiple tools in our suite. Petsakou et al used *Drosophila* as the model system to study intestinal regeneration (24) and as part of this study, generated a single-nuclei profiling data from dissected adult *Drosophila* guts (NCBI GEO Accession GSE218641). For such a data set, a first step might be to compare results to previously reported single cell or single nucleus RNA-seq data (scRNA-seq data). To do this, we compared the top marker genes from each cluster identified in the Petsakou et al. study with the top marker genes from a previously published scRNA-seq analysis of the adult *Drosophila* gut (38) using the ‘Multiple Gene List Enrichment’ option in the ‘Enrichment’ tab at DRscDB. The resulting heatmap of enrichment results shows strong overlap of marker genes between corresponding cell types from the two studies (Supplementary Figure S1A). This shows that a user can use DRscDB to quickly annotate cell types when a new scRNA-Seq dataset is generated. To further investigate biological changes in intestinal epithelial cells (enterocytes) during the recovery stage, we next collected the differentially expressing genes (DEGs) from various clusters of enterocytes and did gene set enrichment analysis (GSEA) using PANGEA. As a gene set, we selected the ‘biological process’ (BP) annotations from gene ontology slim subset (SLIM2), which is a subset assembled by FlyBase to best represent the biological aspects for *Drosophila* (17) (i.e. at the PANGEA search page, we chose ‘SLIM2 GP BP’ under the subheading ‘Drosophila GO Subsets (GO slim)’ in the ‘Gene Ontology Subsets’ sections). The resulting terms that score as over-represented among the DEGs include ‘epithelium development,’ ‘response to external stimulus’ and ‘intracellular signal transduction’, consistent with the findings presented in the study. We next used the node graph function at PANGEA to build an example of a gene-node network that shows what genes drive enrichment of each gene set and shows the overlap of genes that contribute to over-representation of these three biological processes (Supplementary Figure S1B). From this analysis and visualization, we can identify three genes (*InR, hid* and *Src64B*) involved in all three processes and eight genes (*Dsp1, fog, mew, cher, ImpL2, bnl, Socs36E* and *CG43658*) involved in two of the three biological processes. Thus, PANGEA can help users not only to identify and validate biological themes underlining the data but also to prioritize genes in the data set and make testable hypotheses that guide follow-up experiments. As a further analysis for this use case, we also analyzed cell-cell communication events using FlyPhone, with the gene2cell matrix and metadata files (NCBI GEO Accession GSE218641) as inputs and identified strong Notch signaling among ISC/EB cells, which is well supported in the literature (39,40) (Supplementary Figure S1C). This suggests that for other datasets, FlyPhone can be used to validate established signaling events and identify potential new ones.

## Overview of gene- and reagent-centric resources

**Figure 3.**
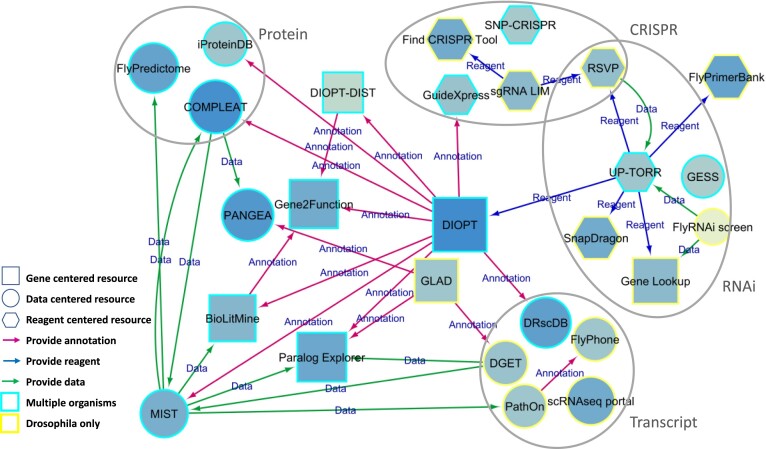
Network view of DRSC online tools. Our online tools can be broadly categorized as supporting reagent, gene, or data-centric information, and as related to protein, DNA or RNA information. While each resource has a unique focus, the majority of the tools are connected by sharing the information either in gene annotation, reagent design and/or datasets. Shape of node represents resource focus while node size and color reflect the tool usage (log_10_ of total access count between 1 August 2023 and 1 August 2024). The border color of nodes represents the species coverage, with blue indicating support of multiple species and yellow indicating support for *Drosophila* only. The edge color represents the information type shared between the tools: red, annotation; green. data; blue. reagent. For example, our ortholog mapping resource DIOPT is used for ortholog mapping at all relevant tools, and an annotation-focused tool covering multiple species.

The most-used online resource in our suite of tools is the DRSC Integrative Ortholog Prediction Tool (DIOPT) (6). DIOPT integrates results from multiple ortholog prediction algorithms at a single interface that supports ortholog mapping among 12 species and can be queried in single-gene or batch mode. In addition, many other DRSC tools have use DIOPT as the core component for cross-species gene searches, e.g. at Gene2Function (5), or cross-species data analysis, e.g. at iProteinDB (41) (Figure 3). At iProteinDB, orthologs from insects and vertebrates are aligned based on DIOPT mapping, and results of phospho-proteomics analyses are annotated on multiple sequence alignments of orthologous genes. DIOPT is also a source of ortholog mapping at resources from other groups, including FlyBase (9,42), the Alliance for Genome Resources (10) and MARRVEL (7). Since our last update, we have developed two additional new resources based on DIOPT.

***Paralog Explorer:*** Paralog Explorer (20) contains paralog relationships from DIOPT and displays results along with additional information relevant to paralog-based studies. The importance of viewing and mining paralog information has been amplified by the new availability of scalable approaches such as CRISPR-Cas methods for simultaneous perturbation of paralogs, an approach useful to address functional redundancy (see for example (43)). Paralog Explorer allows researchers to identify candidate paralogs in the genomes of common genetic model organisms and view co-expression data, protein-protein interaction data, genetic interactions, gene ontology annotations, and phenotype annotations for each paralog. Recent technological advancements have made it possible to efficiently screen for redundant gene functions in cell lines and *in vivo* in *Drosophila*, such as by using multi-target CRISPR arrays and/or by performing synthetic lethal screens. We anticipate that Paralog Explorer can help predict which paralogous genes might act redundantly, thereby assisting in efficient design of such experiments.

***GuideXpress*:**The other new DIOPT-based, gene/reagent-focused resource we added recently is GuideXpress, which was developed to support studies in arthropod disease vector species, including CRISPR pooled screens in mosquito cell lines (8). Many arthropods, such as mosquitos and ticks, can transmit diseases to humans and other animals during a blood meal. Informatics resources developed to support the studies in these species are limited. We adapted the DIOPT pipeline to map orthologs among several arthropod disease vector species, African malaria mosquitos (*Anopheles gambiae*), Asian tiger mosquitos (*Aedes albopictus*), yellow fever mosquitos (*Aedes aegypti*), and black-legged tick or deer tick (*Ixodes scapularis*), as well as *Drosophila*. We also implemented a CRISPR sgRNA design pipeline to identify Cas9 sgRNA target sites for gene knockout at genome-wide scale for three *Anopheles* mosquito species, two *Aedes* mosquito species, the *Aedes* C6/36 cell line genome and *I. scapularis*. Both the ortholog mapping information and the sgRNA design resource can be accessed at the GuideXpress user interface.

## Discussion and conclusions

In the four years since our last update, we have maintained and expanded the FlyRNAi database, continuing to capitalize on the utility of our DIOPT approach and our experience with *Drosophila* and other genetic model systems, and extending in new directions. One area of particular focus was to upgrade and implement new tools and functionality for data handling of scRNA-seq data. Another area has been to extend our resources to include coverage of a group of organisms of increasing public health concern, namely, arthropod vectors of human diseases. In the future, we expect to continue to work in these two areas, as well as to expand our resources to include more data types and develop more AI-driven tools.

## Supplementary Material

gkae917_Supplemental_File

## Data Availability

FlyRNAi is freely available at https://fgr.hms.harvard.edu/tools.
