# Supplementary material for: FlyRNAi.org 2025 update—expanded resources for new technologies and species
Source: Nucleic Acids Res. 2024 Oct 22;53(D1):D958–65. doi: 10.1093/nar/gkae917 (PMC11701652; doi:10.1093/nar/gkae917)

**Supplemental Figure 1. Example outputs for a use-case scRNA-seq dataset analyzed using new data-centric tools.** The single nucleotide RNA-Seq dataset from the publication “Cholinergic neurons trigger epithelial Ca2+ currents to heal the gut” (Petsakou et al, Nature. 2023 PMID: 37722602) was used. The raw data files, the gene2cell expression matrix, and metadata files associated with this study are available at NCBI GEO (Accession ID GSE218641). **A**. Cell-type specific markers (adjusted P value < 0.05 and log2 fold-change >1) for each cluster were compared with clusters from an earlier published study using DRscDB. The resulting output demonstrates the utility of DRscDB for annotating cell types when a new data set is generated. **B**. Differentially expressing genes (DEGs) from all EC clusters were collected and GSEA was performed using PANGEA. The ‘biological process’ (BP) annotation gene set from gene ontology (GO) SLIM2 was selected. Among the significantly enriched biological processes, three terms relevant to this study were selected and visualized using the gene set node graph option. This example demonstrates the utility of PANGEA to interpret and validate the underlining biology within a gene list and the usefulness of PANGEA to generate new hypotheses for follow-up studies. **C**. Cell-to-cell communication events were analyzed by FlyPhone using the gene2cell expression matrix and metadata file as inputs. The top-scoring communication event identified by FlyPhone is the Notch signaling pathway within ISC-EB, consistent with expectation based on the established literature.

**A.**


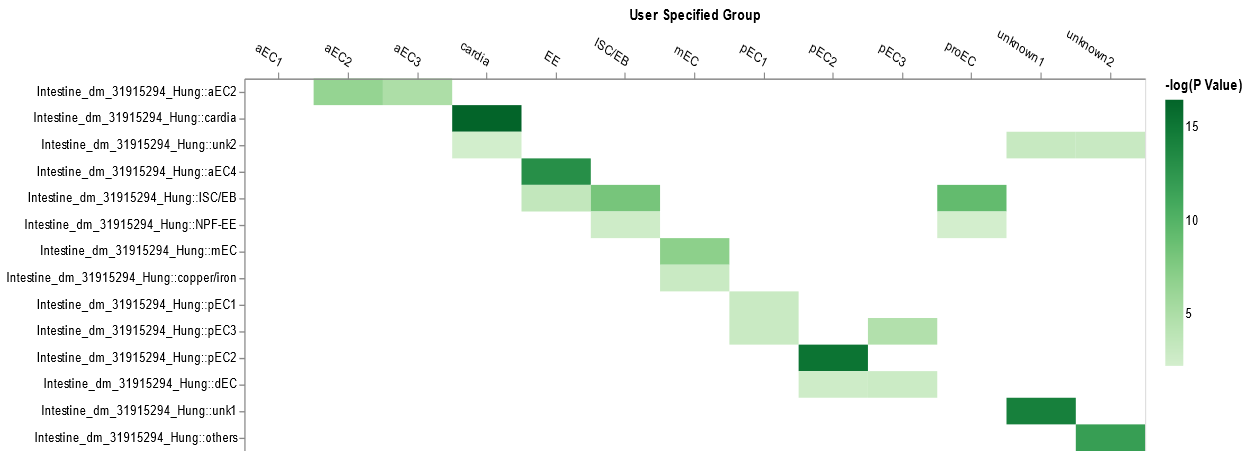


**B.**


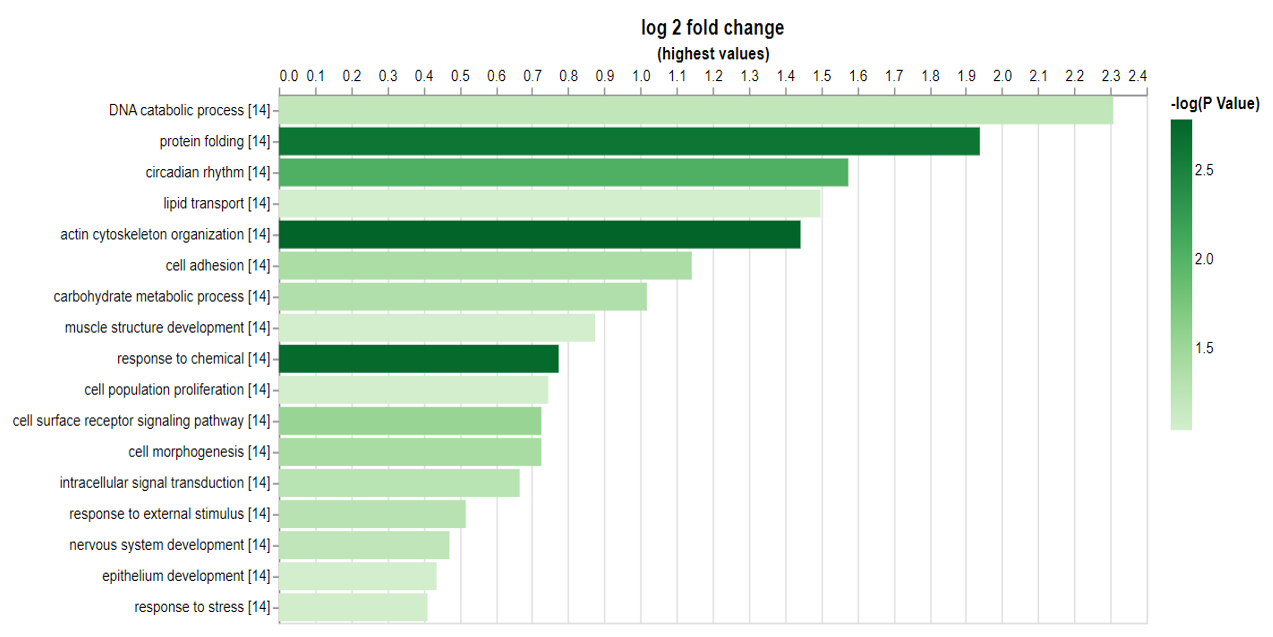


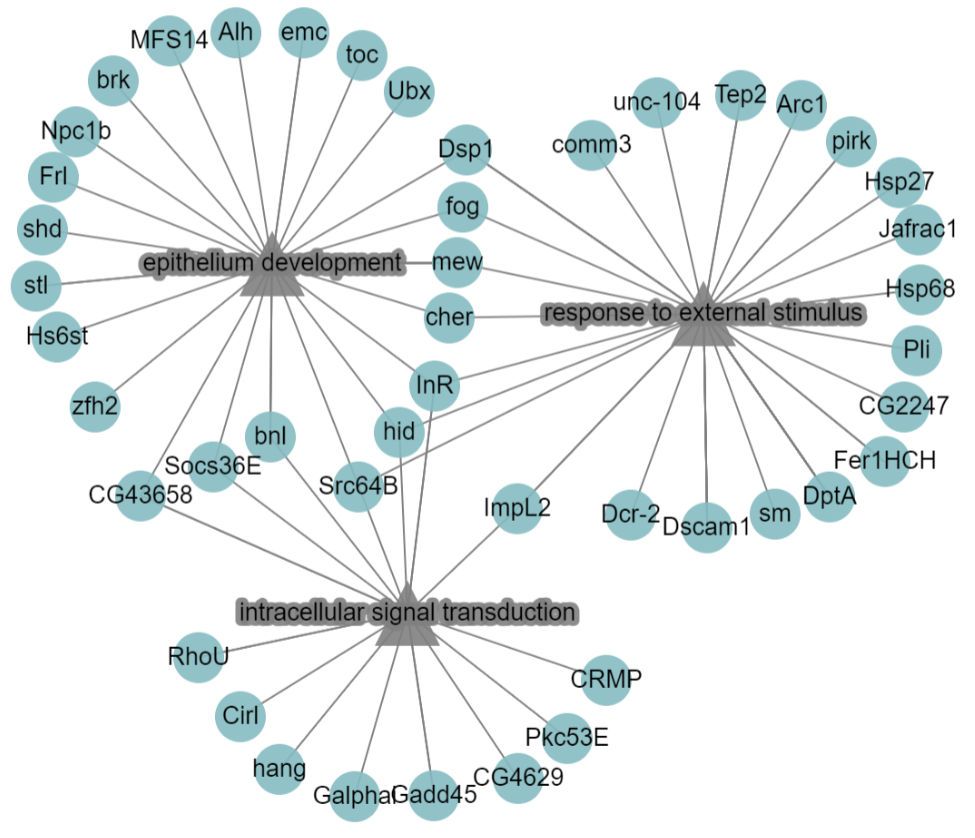


**C.**


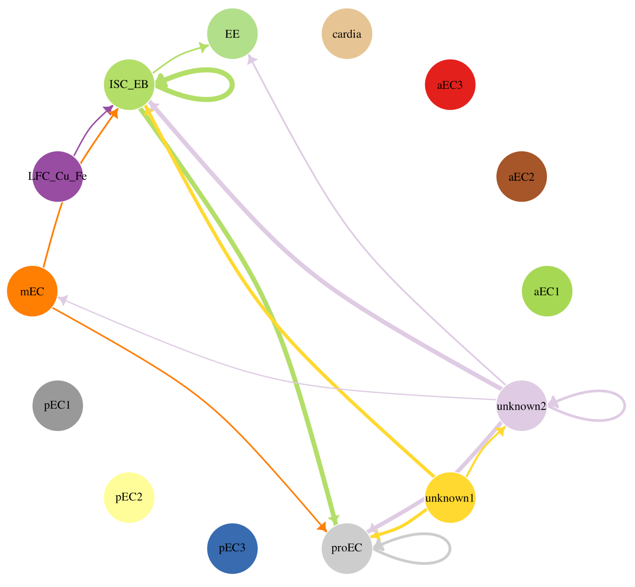


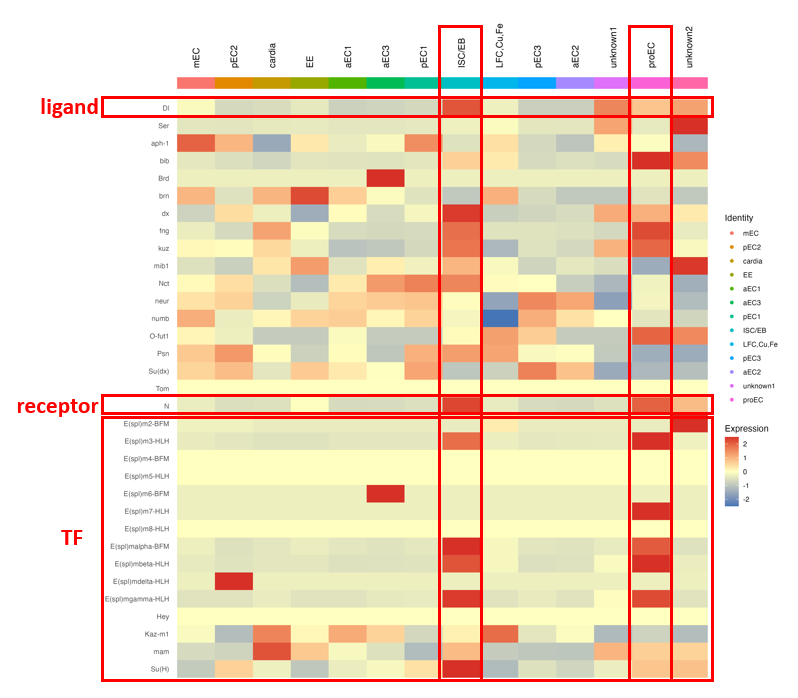

Supplement: gkae917_Supplemental_File [file gkae917_supplemental_file.docx]
